# Melatonin Attenuates Methotrexate-Induced Reduction of Antioxidant Activity Related to Decreases of Neurogenesis in Adult Rat Hippocampus and Prefrontal Cortex

**DOI:** 10.1155/2022/1596362

**Published:** 2022-07-15

**Authors:** Kornrawee Suwannakot, Nataya Sritawan, Salinee Naewla, Anusara Aranarochana, Apiwat Sirichoat, Wanassanun Pannangrong, Peter Wigmore, Jariya Umka Welbat

**Affiliations:** ^1^Department of Anatomy, Faculty of Medicine, Khon Kaen University, Khon Kaen 40002, Thailand; ^2^Neurogenesis Research Group, Department of Anatomy, Faculty of Medicine, Khon Kaen University, Khon Kaen 40002, Thailand; ^3^Department of Basic Medical Science, Faculty of Medicine Vajira Hospital, Navamindradhiraj University, Bangkok, Thailand; ^4^School of Life Sciences, Medical School, Queen's Medical Centre, University of Nottingham, Nottingham NG7 2RD, UK

## Abstract

Previous studies have revealed that the side effects of anticancer drugs induce a decrease of neurogenesis. Methotrexate (MTX), one of anticancer drugs, can induce lipid peroxidation as an indicator of oxidative stress in the brain. Melatonin has been presented as an antioxidant that can prevent oxidative stress-induced neuronal damage via the activation of antioxidant enzymes associated with the increase of neurogenesis. The aims of the present study are to examine the neuroprotective effect of melatonin on the neurotoxicity of MTX on neurogenesis and the changes of protein expression and antioxidant enzyme levels in adult rat hippocampus and prefrontal cortex (PFC). Male Sprague-Dawley rats were assigned into four groups: vehicle, MTX, melatonin, and melatonin+MTX groups. The vehicle group received saline solution and 10% ethanol solution, whereas the experimental groups received MTX (75 mg/kg, i.v.) and melatonin (8 mg/kg, i.p.) treatments. After the animal examination, the brains were removed for p21 immunofluorescence staining. The hippocampus and PFC were harvested for Western blot analysis and biochemical assessments of malondialdehyde (MDA), catalase (CAT), glutathione peroxidase (GPX), and superoxide dismutase (SOD). The immunofluorescence result showed that coadministration with melatonin diminished p21-positive cells in the hippocampal dentate gyrus, indicating a decrease of cell cycle arrest. Melatonin reduced the levels of MDA and prevented the decline of antioxidant enzyme activities in rats receiving MTX. In the melatonin+MTX group, the protein expression results showed that melatonin treatment significantly upregulated synaptic plasticity and an immature neuron marker through enhancing brain derived neurotrophic factor (BDNF) and doublecortin (DCX), respectively. Moreover, melatonin ameliorated the antioxidant defense system by improving the nuclear factor erythroid 2-related factor 2 (Nrf2) in rats receiving MTX. These findings suggested that the effects of melatonin can ameliorate MTX toxicity by several mechanisms, including an increase of endogenous antioxidants and neurogenesis in adult rat hippocampus and PFC.

## 1. Introduction

The anticancer agents have been found to have various adverse effects in several organs [1–4]. Previous studies have found that “Chemobrain” described as the cognitive alterations in the brain can occur in cancer patients receiving anticancer drugs [5, 6]. Methotrexate (MTX) is one of anticancer drugs classified in the antimetabolite group and readily crosses the blood-brain barrier (BBB). The mechanism and action of MTX function through inhibiting thymidylate synthase (TS) and blocking deoxythymidine monophosphate (dTMP) synthesis, resulting in an inhibition of DNA synthesis [7]. Interestingly, several studies have previously postulated the neurotoxicity of MTX in the brain [8–12]. In animal models, MTX-induced degeneration of neurons in the subgranular zone (SGZ) in the dentate gyrus of hippocampus linked to impairment of spatial and recognition memories. These memories were controlled by the hippocampus and PFC, respectively [10–12]. Accumulation of oxidative stress is an essential process that correlates with the degeneration of newly born neurons, whereas enzymatic antioxidants function as free radical scavengers that act as the major source of protection against oxidative stress [13]. Several studies have shown that hippocampal neurogenesis in the SGZ of the dentate gyrus has a clear correlation with oxidative stress and antioxidant enzyme levels [14–17]. A recent study has demonstrated that MTX markedly induces oxidative stress and reduces antioxidant enzyme levels in the hippocampus and PFC [18]. Moreover, MTX treatment causes cell cycle arrest in proliferating cells in the SGZ in the hippocampal dentate gyrus [18]. Therefore, it is believed that exposure to MTX can reduce neurogenesis, and this is closely related to memory deficits.

Various studies have found that antioxidant exposure can stimulate adult neurogenesis in animal models [19–21]. Melatonin (N-acetyl-5-methoxytryptamine) has outstanding properties as an antioxidant and is widely used in experimental studies examining the hippocampus and PFC [22–25]. It is well known that melatonin functions directly as a free radical scavenger and indirectly as reducing oxidative stress through the stimulation of antioxidant enzymes [26, 27]. This hypothesis was supported by an important role of melatonin in encouraging Nrf2 that plays an influential capacity in antioxidant defense systems [14, 17, 28, 29]. In addition, the benefit of melatonin has been shown to promote DCX functions in the hippocampus, which has the potential for neuronal migration and is normally known as markers of premature neurons [11, 30–34]. The regulative function of melatonin in adult neurogenesis is modulated by neurotrophic factors, for example, BDNF, which plays a crucial role in synaptic plasticity and memories [17, 25, 35]. As shown in the previous studies, melatonin ameliorated memory deficits in both spatial and recognition memories and improved the BDNF levels in the adult rat hippocampus [17, 36].

In addition to the interesting studies in the effects of melatonin on memory and neurogenesis, the present study focused on neuroprotective effects of melatonin on molecular mechanism related to neurogenesis in the MTX-induced rats. Therefore, the present study examined that melatonin administration mitigated MTX-induced cell cycle arrest, oxidative stress, and a decline of antioxidant enzymes and levels of DCX, Nrf2, and BDNF protein expression in hippocampus and PFC. However, these side effects of MTX were alleviated by cotreatment with melatonin.

## 2. Materials and Methods

### 2.1. Animals

Fifty-six male Sprague-Dawley rats (7 weeks old, 180-200 g) were acquired from Nomura Siam International, Bangkok, Thailand. Rats were acclimatized in cages and freely accessed water and food during the experiment. Rats were housed under humidity control (30%-60%) in 12 h light/12 h dark cycle (06 : 00 am.-06 : 00 pm.) at room temperature (23 ± 2°C). The experimental procedures were performed in accordance with the Khon Kaen University Ethics Committee in Animal Research (Record No. IACUC-KKU-2/62, approval date: 17 January 2019).

### 2.2. Drug Preparation

Melatonin (Sigma-Aldrich, St. Louis, MO, USA) was immediately dissolved in 10% ethanol solution (RCI Labscan, Bangkok, Thailand) and kept in a falcon tube wrapped with aluminum foil to prevent light-induced melatonin deterioration. 10% ethanol solution was chosen according to previous studies without any toxicity [33, 37, 38]. MTX and leucovorin (LCV) were purchased from Pharmachemie B.V., Haarlem, Netherlands. LCV was used to reduce toxicity of MTX [8, 39].

### 2.3. Experimental Procedures

**Figure 1:**
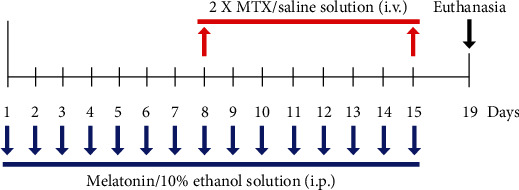
The animal drug treatment schedule. Each MTX/saline solution i.v. injection is represented by the red arrows. The period of melatonin/10% ethanol solution i.p. injections is represented by the blue arrows. Rats were terminated, and the brains were collected at the end of experiment on day 19 depicted by the black arrow.

Rats were randomly arranged into four groups (*n* = 14 animals/group): vehicle, MTX, melatonin, and melatonin+MTX. In the vehicle group, 10% ethanol solution (10% ethanol diluted in saline solution) was given by intraperitoneal (i.p.) injection to rats at 7 : 00 pm. from day 1 to day 15, and a dose of saline solution (0.9% NaCl, Thai Nakorn Patana Co., Ltd., Nonthaburi, Thailand) was administered by intravenous (i.v.) injection at 10 : 00 am. on days 8 and 15 of the experiment. In the melatonin group, melatonin (8 mg/kg) was given by i.p. injection to rats at 7 : 00 pm. from day 1 to day 15. In the MTX group, a dose of MTX (75 mg/kg) was given by i.v. injection at 10 : 00 am. on days 8 and 15 of the experiment. After each MTX injection, LCV (6 mg/kg, at hour 18 h, and 3 mg/kg, at hour 26, 42, and 50) was administered to rats, whereas rats in the vehicle group received an identical volume of saline solution by i.p. injection. Rats in the melatonin+MTX group received equal volumes of melatonin and MTX at the corresponding time points (Figure 1).

### 2.4. Tissue Sample Preparation

On day 3 after the end of the experimental duration, the rats in each group were euthanized by rapid stunning and decapitated. For p21 immunofluorescence study, a cerebral hemisphere was immediately harvested and immersed in 30% sucrose solution (Ajax Finechem, Auckland, New Zealand) for 3 h at 4°C [20]. Next, an optimal cutting temperature (OCT) compound (Sakura Finetek, Torrance, CA, USA) was used to embed the brains, and followed by immediate transferring, the embedded brains to liquid nitrogen cooled isopentane (Sigma-Aldrich, St. Louis, MO, USA) [40]. The other left and right cerebral hemispheres of the hippocampus and PFC were harvested and immediately transferred to liquid nitrogen for Western blotting and biochemical examinations. All tissue samples were transferred to a -80°C freezer until analysis.

### 2.5. Biochemical Assessments

The cold deionized water was used to homogenize the hippocampal and PFC tissues, and the tissue homogenates were centrifuged at 13,000 rpm at 4°C for 10 min. The supernatant was gently collected, and the protein concentrations were analyzed using the NanoDrop spectrophotometer (NanoDrop™ 2000, Thermo Fisher Scientific, USA). All biochemical assessments were detected using the microplate reader (Tecan, Sunrise, Austria) and examined according to the procedures reported previously [17].

#### 2.5.1. Malondialdehyde (MDA) Assay

The evaluation of MDA was carried out to determine lipid peroxidation. The standard solution was prepared using 1,1,3,3-tetraethoxypropane (Sigma-Aldrich, MO, USA) to produce a calibration line. The supernatant was mixed with 20% acetic acid solutions (RCI Labscan, Bangkok, Thailand), 8.1% sodium dodecyl sulfate (Loba Chemie, Mumbai, India), and 0.8% thiobarbituric acid (Sigma-Aldrich, MO, USA). The reaction mixture was boiled in a water bath at 95°C for 1 h before mixing with pyridine (Loba Chemie, Mumbai, India) and n-butanol (RCI Labscan, Bangkok, Thailand). After centrifugation at 4,000 rpm for 10 min, each sample was evaluated at 540 nm, and the measurement was repeated in triplicate.

#### 2.5.2. Superoxide Dismutase (SOD) Assay

A standard curve was prepared using 100 units/mL of SOD enzyme solution (Sigma-Aldrich, MO, USA) as a standard compound. SOD levels were determined by adding the supernatant into the reaction cocktail (pH 7.8) containing 0.108 mM xanthine solutions (Sigma-Aldrich, MO, USA), 216 mM potassium phosphate buffer (pH 7.8, Ajax Finechem, Auckland, New Zealand), 1.1 mM cytochrome C (Sigma-Aldrich, MO, USA), and 10.7 mM ethylenediaminetatraacetic acid (Sigma-Aldrich, MO, USA). After that, the reaction mixture was reacted with 0.1 units/mL of xanthine oxidase enzyme solution (Sigma-Aldrich, MO, USA) before analyzing the absorbance at 540 nm at 0 and 5 min in triplicate.

#### 2.5.3. Catalase (CAT) Assay

1000 units/mL of CAT enzyme solution (Sigma-Aldrich, MO, USA) was prepared as a standard compound. The supernatant was added to the solutions such as 50 mM potassium phosphate buffer, pH 7.0 (Ajax Finechem, Auckland, New Zealand), 30% hydrogen peroxide (Merck, Darmstadt, Germany), and sulfuric acid (RCI Labscan, Bangkok, Thailand). The reaction compound was incubated in the dark condition for 10 min before reacted with potassium permanganate solution (pH 7.0, Ajax Finechem, Auckland, New Zealand). The concentration of CAT was detected by evaluating the absorbance at 540 nm, and each sample was performed in triplicate.

#### 2.5.4. Glutathione Peroxidase (GPX) Assay

The solution of GPX standard was performed by 20 units/mL of GPX enzyme solution (Sigma-Aldrich, MO, USA). GPX levels were examined by mixing the supernatant with the reaction cocktail (pH 7.0), including, *β*-nicotinamide adenine dinucleotide phosphate (Sigma-Aldrich, MO, USA), sodium azide (Sigma-Aldrich, MO, USA), L-glutathione oxidized (Sigma-Aldrich, MO, USA), glutathione reductase solutions (Sigma-Aldrich, MO, USA), and sodium phosphate buffer, pH 7.0 (Qrec, New Zealand). The reaction mixture was mixed with 5,5′-dithiobis-(2-nitrobenzoic acid) (Sigma-Aldrich, MO, USA) before incubating in the dark for 10 min. After that, 30% hydrogen peroxide solution and the reaction compound were mixed together, and the absorbance was detected at 405 nm in triplicate.

### 2.6. Western Blot Analysis

The hippocampus and PFC were homogenized on ice with cold lysis buffer contained protease inhibitors (Sigma-Aldrich, St. Louis, MO, USA) [41]. After centrifugation for 10 min, total protein was quantified with the Lowry method [1]. Equal protein amounts (30 *μ*g) per lane were separated on sodium dodecyl sulfate-polyacrylamide gel electrophoresis (SDS-PAGE), and the separated proteins were blotted onto nitrocellulose membranes (GE Healthcare Life Sciences, Freiburg, Germany). Membranes were blocked with 5% bovine serum albumin (Merck, Millipore, MA, USA) in Tris-buffered saline/0.1% Tween 20 (TBST) for 1 h and incubated at 4°C overnight with the following primary antibodies: anti-Nrf2 (1 : 1000), anti-BDNF (1 : 1000), anti-GAPDH (1 : 20000) (Abcam, Cambridge, UK), and anti-DCX (1 : 200) (Santa Cruz Biotechnology, Dallas, TX, USA). Then the membranes were washed with TBST before probed with anti-mouse (1 : 2000), anti-goat (1 : 2000) and anti-rabbit (1 : 2000) (DAKO, Glostrup, Denmark) for 1 h and examined by enhanced chemiluminescence (ECL) using ECL reagents (GE Healthcare, Buckingham, UK). The images of blots were developed with an Image Quant ECL Imager (GE Healthcare, Chicago, IL), and analyzing the images of protein bands was performed using Image J 1.53a software (Wayne Rasband, National Institutes of Health, USA). Each sample was done in triplicate.

### 2.7. Immunofluorescence Staining of p21

The frozen brains were fixed with 30% sucrose in 0.5% paraformaldehyde solution overnight before serially sectioning into 40 *μ*m along the coronal plane to get the whole dentate gyrus (bregma point -2.3 to -6.3 mm) [42] using a cryostat (Cryostat Series HM550 Microm international, A.S. Science Co., Ltd., Walldorf, Germany) and collected into 24-well plates containing the cryoprotective buffer. Sections were incubated in blocking buffer (20% normal goat serum (Abcam, Cambridge, UK) in PBS containing 0.5% Triton X-100) for 1 h and incubated with an anti-p21 (1 : 100, Santa Cruz Biotechnology, TX, USA) at 4°C overnight. The next day, sections were washed with buffer (PBS containing 0.3% Triton X-100), followed by incubating with an Alexa Fluor 488 rabbit anti-mouse IgG (1 : 300, Invitrogen, San Diego, CA, USA) for 1 h, and were labeled with propidium iodide (PI) (1 : 6000, Sigma-Aldrich, St. Louis, MO, USA). Nine sections from every 8th brain section of the entire length of the dentate gyrus were selected from each rat for staining [43]. The p21-positive cells within the three cells of the internal rim of the dentate gyrus were scored, and a total positive cell count of nine sections from each rat were multiplied by 8 for quantification. Fluorescence images were taken at 10× and 40× magnifications using a fluorescence microscope (Nikon ECLIPSE 80i, Melville, NY, USA).

### 2.8. Statistical Analysis

GraphPad Prism 5.0 (San Diego, CA, USA) was carried out for statistical analysis in this experiment. The results are displayed as mean ± SEM with *p* < 0.05 being considered statistically significant. One-way ANOVA with Bonferroni post hoc test were utilized to compare differences among groups in the analysis of immunofluorescence study, Western blotting, and biochemical assessments.

## 3. Results

### 3.1. Melatonin Attenuates Cell Cycle Arrest Induced by MTX

**Figure 2:**
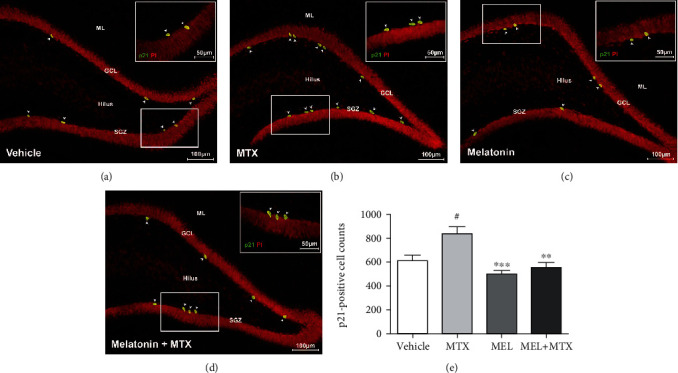
(a–d) Representative immunofluorescence staining of p21. The white arrowheads indicate p21-positive cells (green) in the SGZ of the dentate gyrus. Propidium iodide (PI) staining indicates the nucleus (red). Inserted images are magnified images of p21-positive cells (bar scales: 50 *μ*m, 40×). (e) The histogram showed significantly increased p21-positive cells in the MTX group in comparison with the vehicle group. ^##^*p* < 0.01 vs. the vehicle group. ∗∗*p* < 0.01, ∗∗∗*p* < 0.001 vs. the MTX group. ML: molecular layer; GCL: granule cell layer; SGZ: subgranular zone.

To determine cell cycle arrest induced by MTX, immunofluorescence staining of p21 was performed. The p21-positive cells are detected in the SGZ of the dentate gyrus as shown in Figures 2(a)–2(d). MTX treatment significantly upregulated p21-positive cell numbers in the MTX group in comparison with the vehicle group. Receiving melatonin significantly attenuated the number of p21-positive cells in the MEL+MTX group in comparison with the MTX group. Nevertheless, significant differences in the number of p21-positive cells were not observed between the vehicle and MEL+MTX groups. Data represents mean ± SEM, vehicle: 610.70 ± 49.17 cells; MTX: 841.30 ± 57.44 cells; MEL: 499.20 ± 32.06 cells; and MEL+MTX: 550.70 ± 49.25 cells (*p* < 0.05, *p* < 0.01, *p* < 0.001, Figure 2(e)).

### 3.2. Melatonin Alleviates Oxidative Stress and Promotes Antioxidant Function in the Hippocampus and PFC Induced by MTX

#### 3.2.1. MDA levels

**Figure 3:**
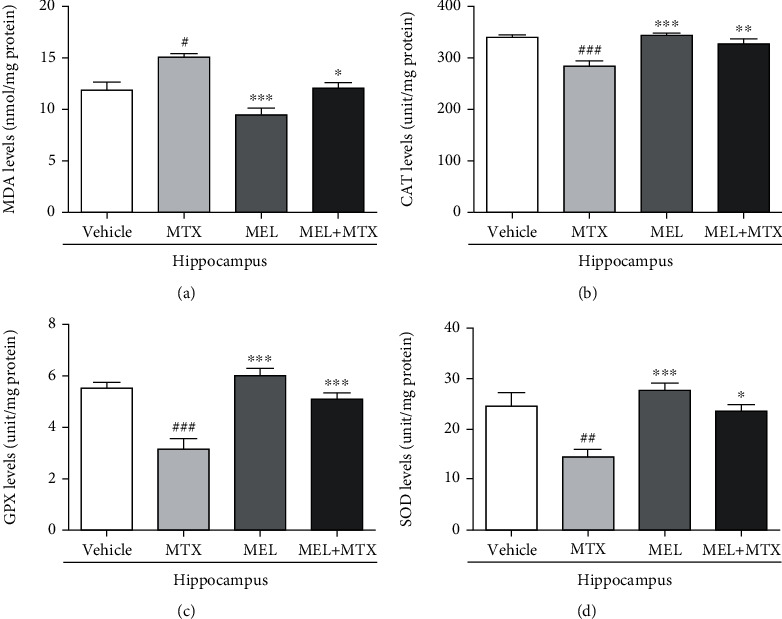
Melatonin ameliorates (a) MDA, (b) CAT, (c) GPX, and (d) SOD levels in the hippocampus of MTX-treated rats. ^#^*p* < 0.05, ^##^*p* < 0.01, and ^###^*p* < 0.001 vs. the vehicle group. ∗*p* < 0.05, ∗∗*p* < 0.01, and ∗∗∗*p* < 0.001 vs. the MTX group. MDA: malondialdehyde; CAT: catalase; GPX: glutathione peroxidase; SOD: superoxide dismutase.

**Figure 4:**
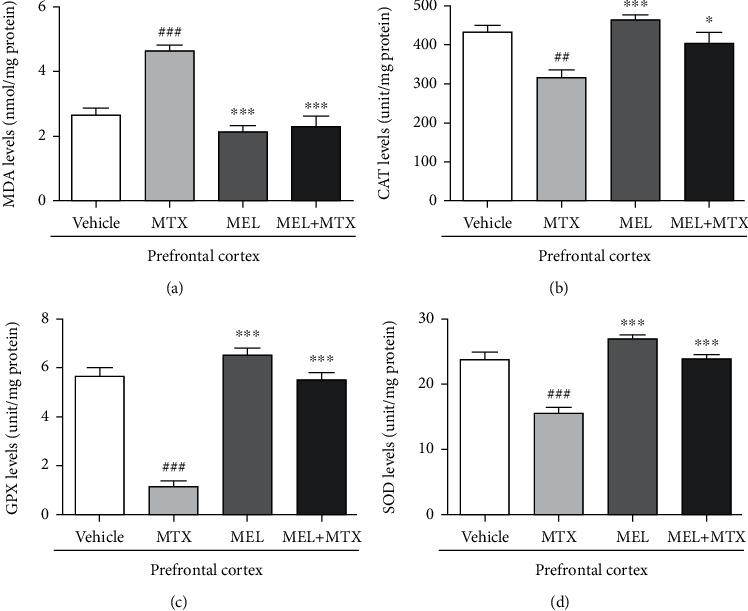
Melatonin improves (a) MDA, (b) CAT, (c) GPX, and (d) SOD levels in the PFC of MTX-induced rats. ^##^*p* < 0.01 and ^###^*p* < 0.001 vs. the vehicle group. ∗*p* < 0.05 and ∗∗∗*p* < 0.001 vs. the MTX group. MDA: malondialdehyde; CAT: catalase; GPX: glutathione peroxidase; SOD: superoxide dismutase.

A significant increase of the MDA level in the hippocampus was detected in the MTX group in comparison with the vehicle group. A significant decline in the MDA level was found in the MEL+MTX group in comparison with the MTX group. Data represents mean ± SEM of the MDA levels in the hippocampus, vehicle: 11.88 ± 0.81 nmol/mg protein; MTX: 15.04 ± 0.38 nmol/mg protein; MEL: 9.47 ± 0.68 nmol/mg protein; and MEL+MTX: 12.05 ± 0.56 nmol/mg protein (*p* < 0.05, *p* < 0.001, Figure 3(a)). In the PFC, the MDA level in the MTX group was significantly greater than the vehicle group. No significant variation was found between the MEL+MTX and vehicle groups. Data represents mean ± SEM of the MDA levels in the PFC, vehicle: 2.62 ± 0.24 nmol/mg protein; MTX: 4.62 ± 0.18 nmol/mg protein; MEL: 2.14 ± 0.19 nmol/mg protein; and MEL+MTX: 2.29 ± 0.32 nmol/mg protein (*p* < 0.001, Figure 4(a)).

#### 3.2.2. CAT Levels

A significant decline of the CAT level in the hippocampus was revealed in the MTX group in comparison with the vehicle group. However, melatonin treatment significantly increased CAT levels in the MEL+MTX group in comparison with the MTX group. Data represents mean ± SEM of the CAT levels in the hippocampus, vehicle: 338.30 ± 6.34 unit/mg protein; MTX: 283.10 ± 10.71 unit/mg protein; MEL: 342.80 ± 4.22 unit/mg protein; and MEL+MTX: 326.40 ± 10.50 unit/mg protein (*p* < 0.01, *p* < 0.001, Figure 3(b)). In the PFC, the CAT levels of animals in the MTX group were significantly decreased in comparison with the vehicle group, whereas no significant differences in the CAT levels were detected between the vehicle and MEL+MTX groups. Data represents mean ± SEM of the CAT levels in the PFC, vehicle: 432.60 ± 17.23 unit/mg protein; MTX: 315.0 ± 20.45 unit/mg protein; MEL: 462.80 ± 13.03 unit/mg protein; and MEL+MTX: 403.30 ± 27.88 unit/mg protein (*p* < 0.05, *p* < 0.01, *p* < 0.001, Figure 4(b)).

#### 3.2.3. GPX Levels

Melatonin treatment mitigated MTX-induced reduction in the GPX level in the hippocampus. In the MEL+MTX group, the levels significantly increased in comparison with the MTX group. Data represents mean ± SEM of the GPX levels in the hippocampus, vehicle: 5.52 ± 0.20 unit/mg protein; MTX: 3.14 ± 0.42 unit/mg protein; MEL: 5.99 ± 0.30 unit/mg protein; and MEL+MTX: 5.09 ± 0.26 unit/mg protein (*p* < 0.001, Figure 3(c)). Moreover, the GPX levels in the PFC of animals in the MEL+MTX group did not significantly differ from the vehicle group, but significantly differed from the MTX group. Data represents mean ± SEM of the GPX levels in the PFC, vehicle: 5.65 ± 0.34 unit/mg protein; MTX: 1.13 ± 0.23 unit/mg protein; MEL: 6.51 ± 0.29 unit/mg protein; and MEL+MTX: 5.49 ± 0.32 unit/mg protein (*p* < 0.001, Figure 4(c)).

#### 3.2.4. SOD Levels

MTX treatment induced the decline of SOD levels in the hippocampus. SOD levels in the MTX group were significantly lower than the vehicle group. Data represents mean ± SEM of the hippocampal SOD levels, vehicle: 24.55 ± 2.69 unit/mg protein; MTX: 14.49 ± 1.57 unit/mg protein; MEL: 27.66 ± 1.41 unit/mg protein; and MEL+MTX: 23.64 ± 1.23 unit/mg protein (*p* < 0.05, *p* < 0.01, *p* < 0.001, Figure 3(d)). Moreover, treatment with melatonin in MTX-treated rats improved SOD levels in the PFC. The SOD levels in the PFC in the MEL+MTX group were significantly greater than the MTX group, but this was not detected in comparison with the vehicle group. Data represents mean ± SEM of the PFC SOD levels, vehicle: 23.73 ± 1.13 unit/mg protein; MTX: 15.53 ± 0.85 unit/mg protein; MEL: 26.93 ± 0.65 unit/mg protein; and MEL+MTX: 23.87 ± 0.62 unit/mg protein (*p* < 0.001, Figure 4(d)).

### 3.3. Melatonin Treatment Reversed the MTX-Induced Decreases of DCX, Nrf2, and BDNF Levels

#### 3.3.1. DCX Levels

**Figure 5:**
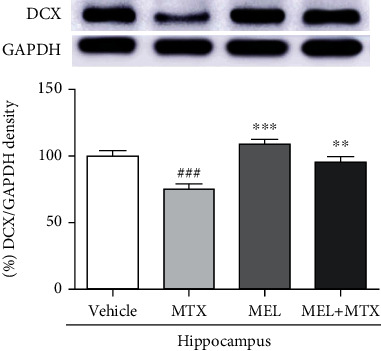
Melatonin improves DCX expression in the hippocampus of MTX-induced rats. ^###^*p* < 0.001 vs. the vehicle group. ∗∗*p* < 0.01 and ∗∗∗*p* < 0.001 vs. the MTX group. DCX: doublecortin.

A significant decrease of DCX expression in the hippocampus was demonstrated in the MTX group in comparison with the vehicle group, while melatonin treatment relieved MTX-suppressed DCX expression. A significant decline of DCX expression in the MEL+MTX group was not found in comparison with the MTX group. Data represents mean ± SEM of DCX levels, vehicle: 99.82 ± 4.076; MTX: 75.14 ± 3.461; MEL: 108.8 ± 3.314; and MEL+MTX: 95.09 ± 4.155 (*p* < 0.01, *p* < 0.001, Figure 5).

#### 3.3.2. Nrf2 Levels

**Figure 6:**
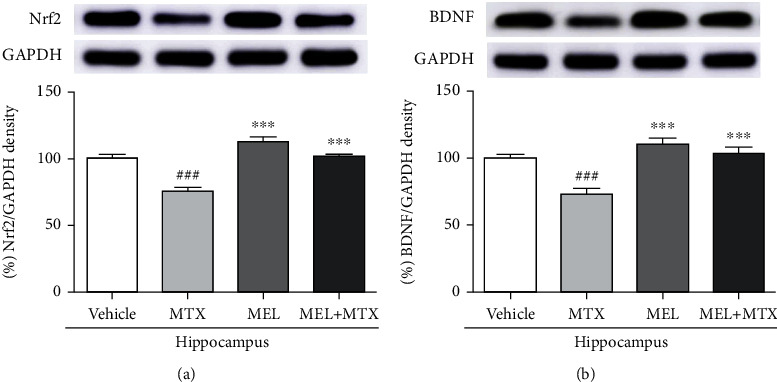
Melatonin restores (a) Nrf2 and (b) BDNF expression in the hippocampus of MTX-induced rats. ^###^*p* < 0.001 vs. the vehicle group. ∗∗∗*p* < 0.001 vs. the MTX group. Nrf2: nuclear factor erythroid 2-related factor 2; BDNF: brain-derived neurotrophic factor.

**Figure 7:**
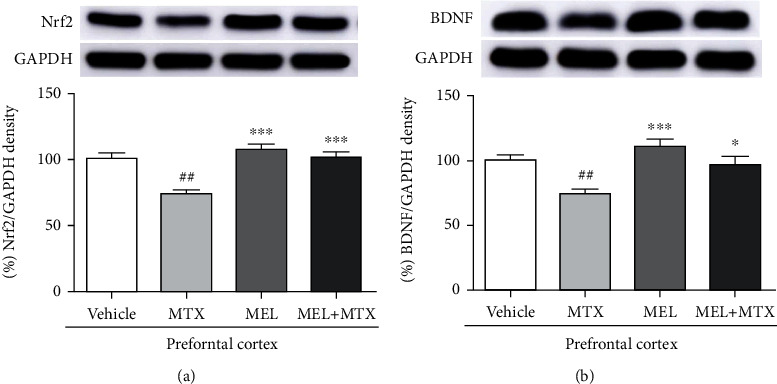
Melatonin enhances (a) Nrf2 and (b) BDNF protein levels in the PFC of MTX-induced rats. ^##^*p* < 0.01 vs. the vehicle group. ∗*p* < 0.05 and ∗∗∗*p* < 0.001 vs. the MTX group. Nrf2: nuclear factor erythroid 2-related factor 2; BDNF: brain-derived neurotrophic factor.

Nrf2 expression of the hippocampus was markedly upregulated in the MEL+MTX group in comparison with the MTX group. Data represents mean ± SEM of Nrf2 levels in the hippocampus, vehicle: 100.80 ± 2.40; MTX: 75.56 ± 3.19; MEL: 112.50 ± 4.47; and MEL+MTX: 101.60 ± 2.02 (*p* < 0.001, Figure 6(a)). In the PFC, there was not a significant difference in the Nrf2 expression when compared between the MEL+MTX and vehicle groups. This result revealed that melatonin conceivably improves the expression of Nrf2 to ordinary levels in MTX-treated rats. Data represents mean ± SEM of Nrf2 levels in the PFC, vehicle: 100.40 ± 4.93; MTX: 73.99 ± 3.22; MEL: 107.60 ± 3.97; and MEL+MTX: 101.30 ± 4.47 (*p* < 0.01, *p* < 0.001, Figure 7(a)).

#### 3.3.3. BDNF Levels

The expression of BDNF level in the hippocampus in the MTX group was significantly less than the vehicle group. However, melatonin treatment significantly restored MTX-induced reduction of BDNF levels in the MEL+MTX group. Data represents mean ± SEM of BDNF levels in the hippocampus, vehicle: 99.76 ± 3.10; MTX: 72.84 ± 3.95; MEL: 110.50 ± 4.26; and MEL+MTX: 103.70 ± 4.33 (*p* < 0.001, Figure 6(b)). Moreover, BDNF expression of the PFC in the MEL+MTX group was not significantly different from the vehicle group but it significantly differed from the MTX group. Data represents mean ± SEM of BDNF levels in the PFC, vehicle:100.60 ± 3.43; MTX: 74.54 ± 2.97; MEL: 110.90 ± 5.81; and MEL+MTX: 96.93 ± 6.25 (*p* < 0.05, *p* < 0.01, *p* < 0.001, Figure 7(b)).

## 4. Discussion

Chemotherapy-induced deterioration of memory and hippocampal neurogenesis has been recently reported [44]. MTX is a chemotherapeutic medication used to treat cancer, neoplastic disorders, and rheumatoid arthritis. However, the use of this drug is restricted because of its side effects on multiple organs such as hepatotoxicity, renal toxicity, and neurotoxicity [3, 4, 8]. The present work aimed to study the adverse effects of MTX on hippocampus and PFC, which are the brain areas that are related to neurogenesis and cognitive functions [45, 46].

Numerous studies suggest that the SGZ of dentate gyrus in the hippocampus is where neurogenesis occurs throughout life [47, 48]. Earlier reports have demonstrated that the toxicity of MTX impaired hippocampal neurogenesis. MTX induces decreases in neurogenesis in the SGZ of the hippocampal dentate gyrus including proliferation and survival of cells and immature neurons using immunofluorescence studies [10–12]. Many previous studies have shown that immature neurons are most commonly characterized by doublecortin or DCX [30, 31]. DCX is a microtubule-associated protein that usually expresses in immature neurons develop into new neurons [49]. In migrating cells, DCX binds to microtubules and results in promoting the movement of neuronal cells [50]. Therefore, DCX is important for neuronal migration by regulating microtubule stabilization [51]. The present study was confirmed more precisely with the protein expression levels. The result shows that the toxicity of MTX significantly reduced the DCX levels in the hippocampus. This is comparable to previous reports that depict that the DCX levels in the hippocampus are dramatically reduced in MTX-treated rats [18, 52]. A previous report demonstrates that melatonin is a pharmacological intervention protecting against chemotherapy-induced neurogenic impairment [44]. The present study clearly shows that melatonin can ameliorate DCX expression levels in the melatonin+MTX group. In line with previous researches, melatonin obviously upregulates the level of immature neurons in the SGZ of the dentate gyrus in adult rats treated with chemotherapy such as MTX and 5-fluorouracil (5-FU) and antiepileptic drug such as valproic acid (VPA) [11, 33, 34]. Moreover, DCX stabilizes microtubules to promote microtubule polymerization, which is necessary for neurite and dendrite formation [49]. Previous studies have reported that melatonin administration in C57BL/6 mice increases DCX-labeled cells especially immature neurons with more mature dendrites in the hippocampal dentate gyrus [30, 31]. Extending more dendritic complexity of new generated neurons into the granule cell layer in the dentate gyrus and then the molecular layer to organize synaptic connection is essential for the integration of new neurons into the hippocampal circuitry [53, 54].

Further study in the present work confirms one of the negative consequences of MTX on neurogenesis in the SGZ of the dentate gyrus is cell cycle arrest using p21 immunofluorescence examination [55]. The p21 is well known as a cyclin-dependent kinase (CDK) inhibitor, which promotes both CDKs inhibition and cycle arrest during the G1/S phase activated by various stimuli [56]. Focus on the present study, an improvement of p21-positive cells in the MTX group indicates upregulation of cell cycle arrest. Corresponding to our previous research, rats receiving MTX showed an increase in p21-positive cells in the SGZ of the hippocampal dentate gyrus [18, 52]. It is concluded that the toxicity of MTX stimulates cell cycle arrest, which has a negative effect on neurogenesis. However, the present research postulates the protective effect of melatonin against MTX-induced cell cycle arrest in the hippocampus. The results demonstrate the decrease of p21-positive cells in the SGZ of hippocampal dentate of rats in the melatonin+MTX group. Our previous study suggested that cotreatment with melatonin in 5-FU-treated rats could relieve the increase of cell cycle arrest [17]. Altogether, melatonin could inhibit oxidative stress caused by side effects of MTX, resulting in the decrease of cell cycle arrest in the hippocampus. Therefore, the present study shows that melatonin can improve neurogenesis and inhibit cell cycle arrest in MTX-treated rats.

Associations between neurogenesis and BDNF have been found in several studies [57, 58]. BDNF is a neurotrophin family of growth factors and plays important activities in several fields of brain functions, for example, cell survival, learning, and memory [58–60]. Signaling pathway of BDNF enhances the cAMP response element binding protein (CREB) transcription factor, which regulates the nerve cell survival and increases synaptic plasticity and neurogenesis [57, 61]. Changes in synaptic plasticity caused by various factors such as age and depression have been investigated [62, 63]. For example, depression reduces synaptic plasticity in the hippocampus via downregulation of synaptic proteins and growth factors. Depression disturbs expression of N-methyl-D-aspartate (NMDA) receptor gene in the PFC, which is very important for controlling synaptic plasticity [63]. In addition, a previous study has reported that fluoxetine, a selective serotonin reuptake inhibitor (SSRI) antidepressant, mitigates memory impairments and depletion of hippocampal neurogenesis in rats caused by VPA associated with decreasing of BDNF levels in the hippocampus [64]. MTX also causes memory impairment in adult rats, and toxicity of MTX causes an impairment of spatial and recognition memories linked to the degeneration of neurons in the SGZ of the dentate gyrus. Spatial and recognition memories are regulated by the hippocampus and PFC, respectively [10–12]. Moreover, our other studies have confirmed that MTX induces a decrease of BDNF level in the adult rat hippocampus and PFC [18, 52]. In line with the present work, the examination using Western blotting assay manifests that MTX can suppress the expression of BDNF in hippocampus and PFC. Here, the relationship in neuroprotective effect of melatonin and BDNF has been previously reported. Melatonin can ameliorate the BDNF levels in the hippocampus and PFC of 5-FU-treated rats [17]. Consistent with the present study, the result demonstrates that melatonin can upregulate BDNF expression in the hippocampus and PFC of rats receiving MTX. These findings demonstrate that the cotreatment with melatonin in MTX-treated rats stimulated the levels of BDNF in both the hippocampus and PFC, which has an important function in neurogenesis and synaptic plasticity related to learning and memory [57, 65].

Several researches have reported the function of oxidative stress in neurodegenerative disorders [66–68]. MTX could upregulate ROS and induce oxidative stress in multiple organs [3, 4, 9, 69]. The present work demonstrated that rats in the MTX group had an accumulation of MDA levels in both hippocampus and PFC. It is well known that MDA is the final product of lipid peroxidation and commonly used as the most popular and dependable markers to determine oxidative stress [70]. Therefore, lipid peroxidation caused by an increment of ROS accumulation leads to cell membrane leakage, resulting in changes of protein structure and function, and DNA damage [71, 72]. Our prior researches have shown that MTX enhances accumulation of MDA levels and decreases in the levels of antioxidant enzymes in the hippocampus and PFC in adult rats [18, 52]. Here, the present experiment found that co-administration with melatonin can decrease the levels of MDA in MTX-treated rats. In the present study, moreover, MTX enhanced an accumulation of oxidative stress that is verified by the reduction of the SOD, CAT, and GPX levels in the hippocampus and PFC. These results reveal that receiving MTX is a high-risk factor to generate oxidative stress and induces the reduction of antioxidant enzymes required to maintain ROS balance in the hippocampus and PFC.

Melatonin has antioxidant capability and acts as a free radical scavenger [26], which directly inhibits reactive oxygen species (ROS) and reactive nitrogen species (RNS) [73]. To restrict overexpression of ROS, both enzymatic and nonenzymatic systems of endogenous antioxidants control ROS balance [74]. Melatonin also diminishes oxidative stress via activating the enzymatic antioxidant defenses including SOD, CAT, and GPX [14, 24, 28]. The present study found that melatonin conceivably improved antioxidant activity in both the hippocampus and PFC in rats receiving MTX. Additionally, increases in antioxidant enzyme levels are related to an increment of Nrf2 expression in both the hippocampus and PFC. The most important biological activity under Nrf2 control is the redox homeostasis in the cells [75]. When cells are under oxidative stress, the mechanism of Nrf2-ARE (antioxidant response element) signaling pathway is responsible for the expression of many antioxidant enzymes such as CAT, SOD, and GPX [76]. These important enzymes actively act via ameliorating oxidative stress and free radicals in the cells [77]. Therefore, Nrf2 is one of the important proteins that regulates antioxidant defense systems and strongly acts as a therapeutic target to treat neurodegenerative disorders related to oxidative stress [78]. A recent work revealed that rats in the melatonin+MTX group showed a significant increase in Nrf2 level in the hippocampus and PFC in comparison with the MTX group. Various studies have demonstrated that the outstanding properties of melatonin strongly enhance Nrf2 functions in several mechanisms to rescue traumatic brain injury and neuroinflammation [28, 75, 79, 80]. Altogether, the discovery from the present work indicates that melatonin markedly exhibits neuroprotective effects, which are mediated by activation of Nrf2 in MTX-induced rats. Therefore, the present study reveals an effective role of Nrf2 correlated with an increase in ROS scavenging enzymes and neurogenesis.

## 5. Conclusion

In conclusion, these findings highlighted the side effects of MTX that are strongly related to oxidative stress-suppressed neurogenesis and induced an increase of cell cycle arrest in the hippocampal dentate gyrus. Here, toxicity of MTX also promoted lipid peroxidation and restrained antioxidant enzyme levels and protein expression of DCX, Nrf2, and BDNF. From further investigations, we found that melatonin had a potential to relieve the side effects of MTX by enhancing the antioxidant enzymes and Nrf2 expression to reduce the cell oxidative stress for maintaining the ROS balance and redox homeostasis in the hippocampus and PFC. Moreover, an impairment of neurogenesis and memories was restored by increasing BDNF expression that regulated cell survival and synaptic plasticity via the neuroprotective effect of melatonin.

## Data Availability

The authors confirm that all data supporting the findings of this research is available in the article.

## Abbreviations

5-FU: 5-fluorouracil
ARE: Antioxidant response element
BBB: Blood-brain barrier
BDNF: Brain-derived neurotrophic factor
CREB: cAMP response element binding protein
CAT: Catalase
CDK: Cyclin-dependent kinase
dTMP: Deoxythymidine monophosphate
DCX: Doublecortin
ECL: Enhanced chemiluminescence
GPX: Glutathione peroxidase
GCL: Granule cell layer
i.p.: Intraperitoneal
i.v.: Intravenous
LCV: Leucovorin
MDA: Malondialdehyde
MTX: Methotrexate
ML: Molecular layer
NMDA: N-methyl-D-aspartate
Nrf2: Nuclear factor erythroid 2-related factor 2
OCT: Optimal cutting temperature
PFC: Prefrontal cortex
PI: Propidium iodide
RNS: Reactive nitrogen species
ROS: Reactive oxygen species
SSRI: Selective serotonin reuptake inhibitor
SDS-PAGE: Sodium dodecyl sulfate-polyacrylamide gel electrophoresis
SGZ: Subgranular zone
SOD: Superoxide dismutase
TBST: Tris-buffered saline/0.1% Tween 20
TS: Thymidylate synthase
VPA: Valproic acid.
